# Clinical Conditions “Suggestive of Progressive Supranuclear Palsy”—Diagnostic Performance

**DOI:** 10.1002/mds.28263

**Published:** 2020-09-11

**Authors:** Max-Joseph Grimm, Gesine Respondek, Maria Stamelou, Thomas Arzberger, Leslie Ferguson, Ellen Gelpi, Armin Giese, Murray Grossman, David J. Irwin, Alexander Pantelyat, Alex Rajput, Sigrun Roeber, John C. van Swieten, Claire Troakes, Wassilios G. Meissner, Christer Nilsson, Ines Piot, Yaroslau Compta, James B. Rowe, Günter U. Höglinger, Movement Disorder Society-Endorsed PSP Study Group

**Affiliations:** 1 Department of Neurology, Technische Universität München, Munich, Germany; 2 German Center for Neurodegenerative Diseases (DZNE), Munich, Germany; 3 Department of Neurology, Hannover Medical School, Hannover, Germany; 4 Parkinson’s Disease and Movement Disorders Department, HYGEIA Hospital, National and Kapodistrian University of Athens, Athens, Greece; 5 First Department of Neurology, Aiginiteion Hospital, National and Kapodistrian University of Athens, Athens, Greece; 6 Department of Neurology, Philipps Universität, Marburg, Germany; 7 Center for Neuropathology and Prion Research, Ludwig-Maximilians-Universität, Munich, Germany; 8 Department of Psychiatry and Psychotherapy, University Hospital, LMU Munich, Munich, Germany; 9 Division of Neurology, Royal University Hospital, University of Saskatchewan, Saskatoon, Saskatchewan, Canada; 10 Neurological Tissue Bank and Neurology Department, Hospital Clínic de Barcelona, Universitat de Barcelona, IDIBAPS, CERCA, Barcelona, Spain; 11 Division of Neuropathology and Neurochemistry, Department of Neurology, Medical University of Vienna, Vienna, Austria; 12 Frontotemporal Degeneration Center, Department of Neurology, University of Pennsylvania, Philadelphia, Pennsylvania, USA; 13 Department of Neurology, Johns Hopkins University, Baltimore, Maryland, USA; 14 Department of Neurology, Erasmus Medical Centre, Rotterdam, The Netherlands; 15 London Neurodegenerative Diseases Brain Bank, Institute of Psychiatry, Psychology and Neuroscience, Kings College London, London, UK; 16 University de Bordeaux, Institut des Maladies Neurodégénératives, CNRS UMR 5293, Bordeaux, France; 17 Service de Neurologie, Hôpital Pellegrin, CHU de Bordeaux, Bordeaux, France; 18 University of Otago, Christchurch, and New Zealand Brain Research Institute, Department Medicine, Christchurch, New Zealand; 19 Department of Clinical Sciences, Division of Neurology, Lund University, Lund, Sweden; 20 Centro de Investigación Biomédica en Red sobre Enfermedades Neurodegenerativas (CIBERNED), Hospital Clínic de Barcelona, Barcelona, Spain; 21 Movement Disorders Unit, Neurology Service, Hospital Clínic de Barcelona. Institute of Neuroscience, University of Barcelona, Barcelona, Spain; 22 Institute of Biomedical Research August Pi i Sunyer (IDIBAPS), Barcelona, Spain; 23 Department of Clinical Neurosciences and Cambridge Centre for Parkinson-Plus, Cambridge University, Cambridge, UK

**Keywords:** progressive supranuclear palsy, clinical diagnostic criteria, autopsy, suggestive, early diagnosis, neuropathology

## Abstract

**Background:**

The Movement Disorder Society diagnostic criteria for progressive supranuclear palsy introduced the diagnostic certainty level “suggestive of progressive supranuclear palsy” for clinical conditions with subtle signs, suggestive of the disease. This category aims at the early identification of patients, in whom the diagnosis may be confirmed as the disease evolves.

**Objective:**

To assess the diagnostic performance of the defined clinical conditions suggestive of progressive supranuclear palsy in an autopsy-confirmed cohort.

**Methods:**

Diagnostic performance of the criteria was analyzed based on retrospective clinical data of 204 autopsy-confirmed patients with progressive supranuclear palsy and 216 patients with other neurological diseases.

**Results:**

The conditions suggestive of progressive supranuclear palsy strongly increased the sensitivity compared to the National Institute of Neurological Disorders and Stroke and Society for Progressive Supranuclear Palsy criteria. Within the first year after symptom onset, 40% of patients with definite progressive supranuclear palsy fulfilled criteria for suggestive of progressive supranuclear palsy. Two-thirds of patients suggestive of progressive supranuclear palsy evolved into probable progressive supranuclear palsy after an average of 3.6 years. Application of the criteria for suggestive of progressive supranuclear palsy reduced the average time to diagnosis from 3.8 to 2.2 years.

**Conclusions:**

Clinical conditions suggestive of progressive supranuclear palsy allow earlier identification of patients likely to evolve into clinically possible or probable progressive supranuclear and to have underlying progressive supranuclear palsy pathology. Further work needs to establish the specificity and positive predictive value of this category in real-life clinical settings, and to develop specific biomarkers that enhance their diagnostic accuracy in early disease stages.

Progressive supranuclear palsy (PSP) is a sporadic, adult-onset neurodegenerative disease with average disease duration of approximately 6–8 years. Characteristic neuropathological findings are intracellular aggregation of hyperphosphorylated tau protein with four microtubule binding repeat domains (4-repeat tau) in oligodendrocytes (coiled bodies), astrocytes (tufted astrocytes), and neurons (neurofibrillary tangles).^1–3^ A definite diagnosis of PSP can only be established currently by postmortem neuropathological brain examination.^1,4^

In 2017, the Movement Disorder Society-endorsed PSP study group provided new clinical diagnostic criteria (MDS-PSP criteria)^5^ on the basis of an extensive evaluation of the published literature and a large clinico-pathological cohort.^5,6^ The diagnostic certainty level “suggestive of PSP” (s.o. PSP) was introduced with the intention of allowing an earlier and more sensitive identification of patients presenting with subtle signs suggestive of PSP that may evolve into clinically possible, probable PSP later in disease course, and ultimately have definite PSP on postmortem examination.^5^

Moreover, PSP predominance types were introduced by the MDS-PSP criteria as part of the clinical diagnosis. Not every predominance type is represented in every certainty level. There are seven predominance types for s.o. PSP, five for possible PSP and four for probable PSP:

- s.o. PSP: PSP-CBS (PSP with predominant corticobasal syndrome), PSP-F (PSP with predominant frontal presentation), PSP-OM (PSP with predominant ocular motor dysfunction), PSP-P (PSP with predominant parkinsonism), PSP-PI (PSP with predominant postural instability), PSP-RS (PSP with Richardson’s syndrome), PSP-SL (PSP with predominant speech/language disorder);
- possible PSP: PSP-CBS, PSP-OM, PSP-PGF (PSP with progressive gait freezing), PSP-RS, PSP-SL; and
- probable PSP: PSP-F, PSP-P, PSP-PGF, PSP-RS.

Thus, many patients will change their predominance type as diagnostic certainty increases.

Three retrospective studies validating the MDS-PSP criteria have been published to date and compared them with previous criteria (NINDS-SPSP).^7–9^ Ali et al^7^ analyzed the overall MDS-PSP criteria in an autopsy-confirmed cohort with 66 PSP patients and 63 other neurodegenerative diseases (corticobasal degeneration [CBD], N = 35; frontotemporal lobar degeneration with TDP pathology [FTLD-TDP], N = 1; glial globular tauopathy [GGT], N = 3; multiple system atrophy [MSA], N = 4; Lewy body disease [LBD], N = 20) and suggested significantly increased sensitivity (87.9% vs 45.5%) and slightly reduced specificity (85.7% vs 90.5%) of the MDS-PSP criteria, as compared to the NINDS-SPSP criteria. In this study, the PSP cohort was divided into an early-stage group with neurological evaluation within 3 years after symptom onset and a late-stage group with neurological evaluation after 3 years of symptom onset. In the early-stage group, the sensitivity for s.o. PSP was 84.4% (N = 45), while in the late-stage group it reached 100% (N = 37). The sensitivity for s.o. PSP of the entire cohort was 87.9% (N = 66).^7^

Another study published by the study group of this paper analyzed the diagnostic performance of the MDS-PSP criteria in an independent cohort of 108 autopsy-confirmed PSP cases and 81 cases with other FTLD pathologies, including syndromes of behavioral variant frontotemporal dementia (bvFTD), non-fluent/agrammatic variant primary progressive aphasia (nfvPPA), and corticobasal syndrome (CBS) confirmed improved sensitivity for the MDS-PSP criteria compared to the NINDS-SPSP criteria (72.2%–100% vs 48.1%–61.1%, depending on the certainty level) with reduced specificity (53.1%–95.1% vs 97.5%–100%, depending on the certainty level).^8^

We previously analyzed sensitivity and specificity of the diagnostic category “probable 4-repeat tauopathies” as part of the MDS-PSP criteria in 195 PSP, 55 CBD, and 161 non-4-repeat tauopathies.^9^ These cases were part of the same cohort analyzed in the current study. High specificity for PSP and CBD pathology suggested that MDS-PSP criteria for “probable 4-repeat tauopathies” are suitable for the recruitment of patients into therapeutic trials targeting 4R-tau.^9^

However, the diagnostic performance of the s.o. PSP category needs further verification. Thus, we aimed to determine the reliability of the s.o. PSP diagnostic category in early identification of patients at risk of progressing into possible or probable PSP during the clinical course, and in prediction of underlying PSP pathology.

Therefore, we analyzed a large, clinically well-described cohort of autopsy-confirmed PSP patients and disease controls for (1) the sensitivity, specificity and positive predictive value (PPV) of s.o. PSP to predict underlying PSP pathology as a function of disease duration, (2) the effect of the exclusion criteria thereon, (3) evolution of patients with s.o. PSP into possible and probable PSP, (4) the impact of s.o. PSP on the overall diagnostic performance of the MDS-PSP criteria as compared to the NINDS-SPSP criteria, and (5) the distribution of PSP-predominance types among s.o. PSP with and without PSP pathology.

## Methods

### Protocol Approvals and Patient Consents

Approval for this work was obtained from the local IRBs and the Ethics committee of the Technical University of Munich, Germany. Prior to death, all donors gave written informed consent for the use of their brain tissue and medical records for research purposes.

### Identification of Cases

Patients with autopsy-confirmed diagnoses of PSP,^10–12^ corticobasal degeneration (CBD),^13^ multiple system atrophy (MSA) with a clinical diagnosis of MSA with predominant parkinsonism (MSA-P),^14^ Lewy body disease (LBD)^15^ with clinical history of Parkinson’s disease (PD), and 4R-tau negative frontotemporal lobar degeneration (non-4RT FTLD)^16^ were identified in the registries of nine collaborating brain banks with expertise in neurodegenerative disorders:

1. Neurobiobank Munich, Center for Neuropathology and Prion Research, Ludwig-Maximilians-University, Munich, Germany.
2. MRC London Neurodegenerative Diseases Brain Bank, King’s College, London, UK.
3. Netherlands Brain Bank, Amsterdam in collaboration with the Department of Neurology, Erasmus Medical Center, Rotterdam, The Netherlands.
4. Neurological Tissue Bank of the Biobanc-Hospital Clinic-IDIBAPS, Barcelona, Spain in collaboration with the Neurology Department of the Hospital Clinic.
5. Centre de Ressources Biologiques-Bordeaux Biothèque Santé, Bordeaux, France.
6. Brain Bank of Region Skane, Department of Pathology, Lund University, Lund, Sweden.
7. Johns Hopkins Medical Institutions (JHMI) Brain Resource Center, Baltimore, USA.
8. Penn Center for Neurodegenerative Disease Research (CNDR) brain bank, University of Pennsylvania, Philadelphia, USA.
9. Brain Bank of the Royal University Hospital, University of Saskatchewan, Canada.

The majority of cases that entered these brain banks originated from tertiary neurological hospitals. The participating brain banks searched their inventories for all cases with an autopsy-confirmed diagnosis of PSP, CBD, LBD, MSA, and non-4RT FTLD. All cases with LBD pathology had received a clinical diagnosis of PD or PD with dementia (PDD) prior to death. All cases with MSA pathology had received a clinical diagnosis of MSA-P prior to death. All other cases were selected by pathology, regardless of their clinical diagnosis. Cases with insufficient clinical data were excluded from analysis. Cases from the same cohort had been included in previous studies.^6,9^

### Data Collection

Detailed clinical information was generated for each patient by retrospective chart review. The following features were systematically abstracted: age of onset (defined as onset of first disease-specific clinical signs), age of death, gender, and presence/absence and year of onset of: abnormal saccades, alien limb phenomenon, Alzheimer’s disease-like deficits, apraxia of limb(s), apraxia of speech, asymmetry at onset, asymmetry (persisting), autonomic dysfunction, bradykinesia, cerebellar signs, cognitive dysfunction, cortical sensory loss, dysarthria, dysphagia, dystonia (axial), dystonia of limb(s), falls, freezing of gait, freezing of speech, frontal dysfunction, hallucinations (spontaneous), hallucinations (L-dopa-induced), levodopa-responsiveness, myoclonus, non-specific visual symptoms, postural instability, non-fluent/agrammatic primary progressive aphasia, pyramidal tract signs, rapid hypophonia, rapid micrographia, rest tremor, rigidity (axial predominant), rigidity (limb predominant), semantic dementia, start hesitation, supranuclear gaze palsy, and tremor. Definitions for these clinical features are given in Table 1. In some cases, the year of onset for a respective feature could not be determined and was therefore recorded as present at final record. The term “end of record” refers to a feature being present after the 9^th^ year or at some point in the disease history without indicated time. Features that were not stated as present in the patientś medical records were considered to be absent.

### Validation of the MDS-PSP and the NINDS-SPSP Criteria

Inclusion and exclusion criteria of the MDS-PSP criteria and the NINDS-SPSP criteria were applied retrospectively to our cohort for each patient and each year after onset of the first disease-associated symptom. For our analysis, we interpreted the term “predominant” used in the context of “impairment of episodic memory,” “autonomic failure,” “visual hallucinations or fluctuations in alertness,” and “multisegmental upper and lower motor neuron signs” in the MDS-PSP exclusion criteria^5^ as present in the first 2 years of disease. The presence or absence of core clinical features allowed the allocation of each patient to a diagnostic certainty level, which comprised probable, possible, or s.o. PSP for the MDS-PSP criteria, and probable or possible PSP for the NINDS-SPSP criteria. The specific predominance type according to the MDS-PSP criteria was identified for each patient for each year after symptom onset. Diagnostic allocations to multiple levels of certainty and predominance type according to the MDS-PSP criteria were reduced by applying the Multiple Allocation eXtinction (MAX) rules.^17^ Patients with persisting multiple allocations after application of the MAX rules were handled as separate entities (eg, PSP-P + PSP-F). Sensitivity, specificity, and PPV for both the MDS-PSP and the NINDS-SPSP criteria were determined and compared for the years 1, 3, 6, and 9 and at final antemortem record. Evolution rates into higher diagnostic certainty levels were assessed for s.o., possible and probable PSP according to the MDS-PSP criteria for each year of disease. Distribution of predominance types among each diagnostic certainty level and among each pathological diagnosis were assessed according to the MDS-PSP criteria.

## Results

### Patient Demographics

A total of 204 patients with autopsy confirmed PSP, and 216 cases with other neurological disease (comprising 55 CBD, 50 MSA-P, 51 PD, and 60 non-4RT FTLD patients) with detailed clinical records were included in our analysis. Non-4RT FTLD comprised 46 patients with TAR DNA-binding protein 43 (TDP-43) pathology, 10 patients with three-repeat (3R)-tau pathology (Pick’s disease), and four patients with fused in sarcoma (FUS) protein pathology. The age of onset of first symptoms in the PSP cohort ranged from 41–91 years with a mean age of 66 years and in the non-4RT cohort from 42–80 years with a mean age of 58 years. Detailed demographic data of all patients is given in Table 1.

### Sensitivity of s.o. PSP

**FIG. 1.**
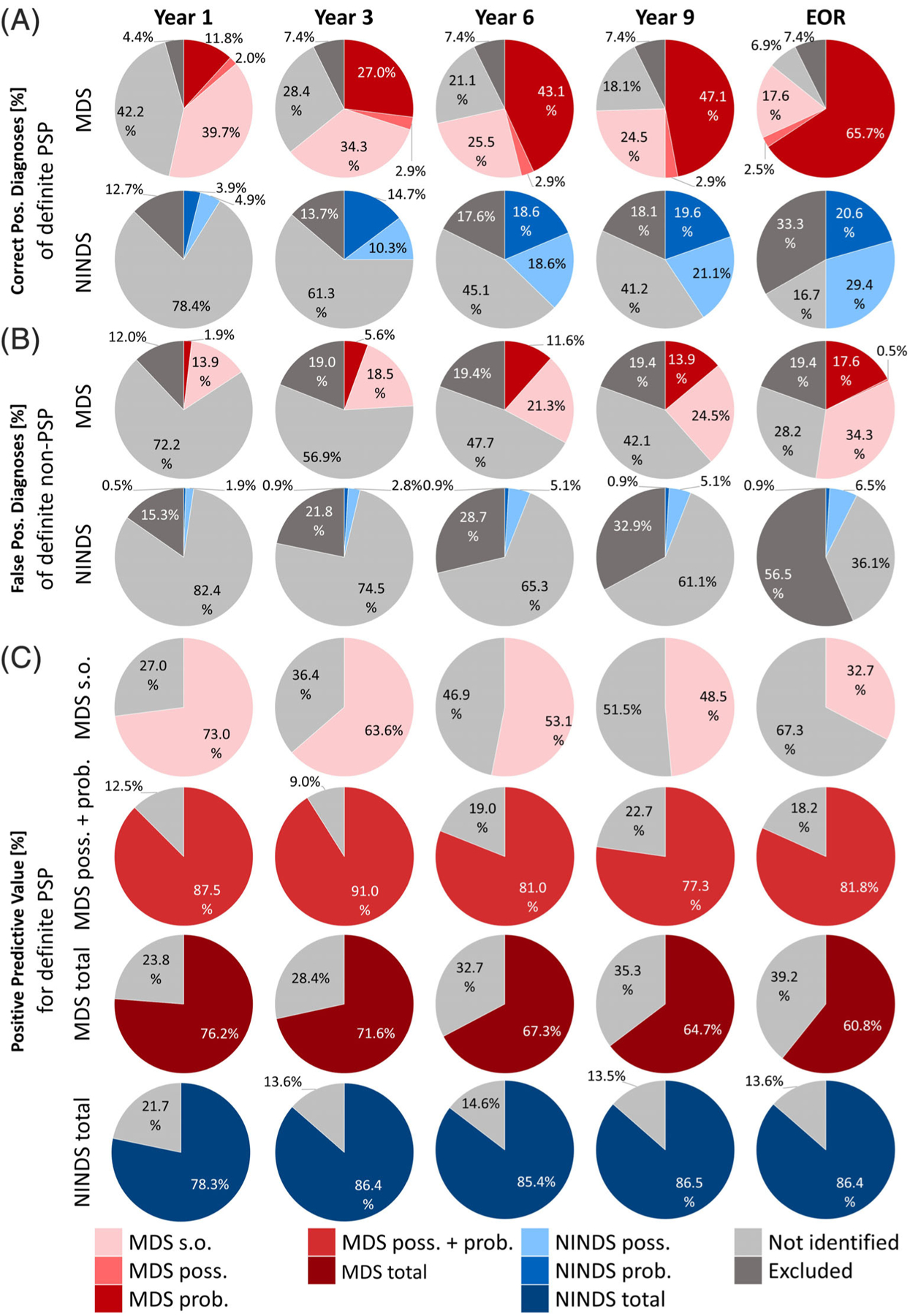
Sensitivity, specificity, and positive predictive value with application of the exclusion criteria. (**A**) Rate of correct positive PSP diagnoses in definite PSP (ie sensitivity) and (**B**) false–positive PSP diagnoses in non-4R-tauopathies (ie, 100%-specificity) with the MDS-PSP criteria (probable, possible or suggestive of PSP) and NINDS-SPSP criteria (probable or possible PSP) as a function of disease duration (1^st^–9^th^ year) since onset of first symptoms. (**C**) Positive predictive value (PPV) for s.o. PSP (first row), possible or probable PSP combined (second row), and PSP of all certainty levels combined (third row) according to the MDS-PSP criteria; and of all certainty levels combined according to the NINDS-SPSP criteria (possible and probable PSP; forth row). Abbreviations: EOR, end of record; Prob., probable PSP; poss., possible PSP; s.o., suggestive of PSP. Not identified = patients not fulfilling the respective clinical diagnostic criteria; excluded = patients meeting the exclusion criteria of the respective clinical diagnostic criteria.

Sensitivity (ie, percentage of correct positive diagnoses from all definite PSP patients) of the retrospectively established diagnosis of s.o. PSP using the MDS-PSP criteria during the course of the disease (1^st^, 3^rd^, 6^th^, 9^th^ year after symptom onset and at final antemortem record) in cases with autopsy-confirmed, definite PSP, after application of the MDS-PSP exclusion criteria, is shown in Figure 1A.

In the 1^st^ year, sensitivity of s.o. PSP was 39.7%, contributing the largest proportion of patients to yield an overall sensitivity of the MDS-PSP criteria of 53.5%.

With increasing disease duration, the sensitivity of s.o. PSP declined (3^rd^ year, 34.3%; 6^th^ year, 25.5%; 9^th^ year, 24.5%; end of record, 17.6%), whereas the overall sensitivity of the MDS-PSP criteria increased (3^rd^ year, 64.2%; 6^th^ year, 71.5%; 9^th^ year, 74.5%; and end of record, 85.8%).

### Contribution of s.o. PSP to the Overall Sensitivity of the MDS-PSP Criteria

The sensitivity of the retrospectively established diagnosis of possible or probable PSP using the MDS-PSP or the NINDS-SPSP criteria during the course of the disease in cases with autopsy-confirmed, definite PSP, after application of the respective exclusion criteria, is also shown in Figure 1A.

Considering only the diagnostic certainty levels of possible and probable PSP, the MDS-PSP criteria yielded slightly higher sensitivity than the NINDS-SPSP criteria throughout the entire disease course (1^st^ year, 13.8% vs 8.8%; 3^rd^ year, 29.9% vs 25.0%; 6^th^ year, 46.0% vs 37.2%; 9^th^ year, 50.0% vs 40.7%; and end of record, 68.2% vs 50.0%).

When the diagnostic certainty level of s.o. PSP was included in the analysis, the MDS-PSP criteria yielded much higher sensitivity than the NINDS-SPSP criteria throughout the entire disease course (1^st^ year 53.5% vs 8.8%, 3^rd^ year 64.2% vs 25.0%, 6^th^ year 71.5% vs 37.2%, 9^th^ year 74.5% vs 40.7%, end of record 85.8% vs 50.0%).

### Effect of the Exclusion Criteria on Sensitivity

We analyzed the effect of correct application versus omission of the exclusion criteria on the sensitivity of the MDS-PSP and NINDS-SPSP criteria (Fig. 1A vs Supplementary Fig. S1A).

The percentage of definite PSP patients fulfilling the exclusion criteria increased with time (MDS-PSP: 1^st^ year 4.4% to 9^th^ year 7.4%; NINDS-SPSP: 1^st^ year 13.7% to 9^th^ year 18.1%; Fig. 1A), indicating that some patients with definite PSP develop clinical features that are not considered as typical for PSP. The fact that this rate was significantly lower in the MDS-PSP than in the NINDS-SPSP criteria reflects the inclusion of a broader phenotypic spectrum in the former, features that were considered as exclusion criteria in the latter criteria.

A total of 7% (N = 15) of definite PSP patients met exclusion criteria of the MDS-PSP criteria within the first 3 years (ie, false–negative cases). The most frequent reason for their exclusion was predominant unexplained autonomic dysfunction in 10 out of 15 cases (67%), followed by impairment of episodic memory suggestive of Alzheimer’s disease in three cases and visual hallucinations in one case. There were two cases with dual pathology, one of them with PSP and LBD and the other one with PSP and AD pathology. Both of them were excluded because of autonomic failure within the first 2 years.

Sensitivity of the s.o. PSP category in the MDS-PSP criteria increased only marginally when the exclusion criteria were omitted (1^st^ year, 39.7% vs 41.7%; 9^th^ year, 24.5% vs 27.0%; Fig. 1A vs Supplementary Fig. S1A).

Overall sensitivity of the MDS-PSP criteria (s.o., possible, and probable) also increased only marginally, when the exclusion criteria were omitted (1^st^ year, 53.5% vs 57.4%; 9^th^ year, 74.5% vs 81.9%; and end of record, 85.8% vs 93.1%; Fig. 1A vs Supplementary Fig. S1A).

The overall sensitivity of the NINDS-SPSP criteria (possible and probable) increased only slightly when the exclusion criteria were omitted (1^st^ year, 8.8% vs 13.8% and 9^th^ year, 40.7% vs 54.0%; Fig. 1A vs Supplementary Fig. S1A). The largest difference in sensitivity when omitting the exclusion criteria could be seen at the end of record (50.0% vs 75.0%; Fig. 1A vs Supplementary Fig. S1A). This was the case because a large number of patients were excluded after the 9^th^ year, or at final record, because symptoms without time specification were assumed to be present at the end of record.

### False–Positive s.o. PSP Diagnoses

False–positive diagnoses of s.o. PSP using the MDS-PSP criteria, after application of the exclusion criteria, in definite non-PSP cases are shown in Figure 1B. As expected, s.o. PSP yielded higher rates of false–positive diagnoses than probable PSP (1^st^ year, 13.9% vs 1.9%; 3^rd^ year, 18.5% vs 5.6%; 6^th^ year, 21.3% vs 11.6%; 9^th^ year, 24.5% vs 13.9%, and end of record, 34.3% vs 17.6%). Surprisingly, possible PSP yielded only one false–positive diagnosis at the end of record in this series.

To identify which patient group would be most prone to misclassification as s.o. PSP (ie, false–positive cases), we analyzed false–positive diagnoses in CBD, MSA, PD, and non-4RT FTLD patients separately (Supplementary Fig. S2, applying exclusion criteria; Supplementary Fig. S3, without applying exclusion criteria).

With the application of the exclusion criteria, most false–positive s.o. PSP diagnoses in the 1^st^ year occurred in the CBD group with 21.8% (Supplementary Fig. S2D), followed by the MSA group with 20% (Supplementary Fig. S2A), and the PD group with 9.8% (Supplementary Fig. S2A). From the 3^rd^ until the 9^th^ year, most false–positive s.o. PSP diagnoses were observed in the MSA group (3^rd^ year and 9^th^ year 30%, Supplementary Fig. S2A). Most of these MSA cases received a diagnosis of s.o. PSP-PI (not shown), which reflects the high prevalence of early postural instability observed in both MSA and PSP. When omitting the exclusion criteria, the MSA group showed the highest prevalence of false–positive s.o. PSP diagnoses from the 1^st^ year (30%) until the 9^th^ year (48%) (Supplementary Fig. S3A), followed by the CBD group from the 1^st^ year (27.3%) until the 9^th^ year (38.2%) (Supplementary Fig. S3D).

At final record, most false–positive s.o. PSP diagnoses were observed in PD cases (58.8% with and 62.7% without applying exclusion criteria), mostly due to presence of the features A3 (“parkinsonism, with tremor and/or asymmetric and/or levodopa responsive”) and C2 (“frontal cognitive/behavioral presentation”) qualifying for s.o. PSP-P.^5^ Second and third most common false–positive s.o. PSP diagnoses occurred in the MSA group (38% with, 56% without exclusion criteria) and in the CBD group (27.3% with, 36.4% without exclusion criteria). The difference between the 9^th^ disease year and final record is based on the fact that the time of onset of some features was not recorded, thus being only considered for the final record analysis.

The rate of false–positive s.o. PSP diagnoses in non-4RT FTLD was 5.0% in the 1^st^ year and continuously increased to 16.7% until the end of record.

In the first 3 years, 13.9% (30/216) non-PSP patients presented with postural instability and therefore qualified for s.o. PSP-PI (not shown). This number was stable until the end of record, because postural instability must occur within the first 3 years to qualify for a diagnosis according to the MDS-PSP criteria. Importantly, however, 7.9% (17/216) were eliminated by the MDS-PSP exclusion criteria. At the end of record, the most frequent s.o. PSP predominance type in non-PSP patients was s.o. PSP-P with 22.7% (49/216), respectively, 19.9% (43/216) after application of exclusion criteria.

### Overall Specificity of the MDS-PSP and the NINDS-SPSP Criteria

Specificity is the percentage to which non-PSP patients were correctly classified as non-PSP (ie, sum of “not identified” and “excluded”). Specificity of the overall MDS-PSP criteria (s.o., possible and probable) was high in the 1^st^ and 3^rd^ year (84.2% and 75.9%) and declined to 61.5% at year 9 (Fig. 1B). Specificity of possible and probable MDS-PSP criteria only (considering s.o. PSP as no diagnosis) was high throughout the clinical course (MDS-PSP: 1^st^ year, 98.1%; 3^rd^ year, 94.4%; and 9^th^ year, 86.1%). NINDS-SPSP criteria maintained a higher specificity throughout the course of disease (1^st^ year, 97.7%; 3^rd^ year, 96.3%; and 9^th^ year, 94.0%; Fig. 1B).

### Effect of the Exclusion Criteria on Specificity

We analyzed the effect of application versus omission of the exclusion criteria on the rate of false–positive diagnoses of the MDS-PSP and NINDS-SPSP criteria (Fig. 1B) vs Supplementary Fig. S1B.

Specificity increased when applying the exclusion criteria, because a number of definite non-PSP patients, which were falsely classified as PSP, were excluded. False–positive diagnoses decreased for the MDS-PSP criteria (1^st^ year s.o. PSP criteria: 19% to 13.9%, overall MDS-PSP criteria: 21.3% to 15.8%) and for the NINDS-SPSP criteria (overall: 4.7% to 2.4%). The elimination of false–positive PSP diagnoses in non-PSP patients was more pronounced in later disease stages (eg, 9^th^ year: s.o. PSP: 33.8% to 24.5%, probable MDS-PSP: 19.4% to13.9%, overall MDS-PSP: 53.2% to 38.4%, overall NINDS-SPSP: 13.5% to 6%).

To identify which differential diagnostic group would benefit most from correct application of the exclusion criteria, we analyzed false–positive diagnoses in MSA, PD, and non-4RT FTLD patients separately (Supplementary Fig. S2, applying exclusion criteria; Supplementary Fig. S3, without applying exclusion criteria). The elimination of false–positive diagnoses by the exclusion criteria was by far strongest for MSA, followed by CBD, then PD, and finally non-4RT FTLD, both for s.o. MDS-PSP, overall MDS-PSP, and overall NINDS-SPSP criteria.

### Positive Predictive Value of MDS-PSP and NINDS-SPSP Criteria

PPVs for the MDS-PSP and the NINDS-SPSP criteria as a function of disease duration are displayed in Figure 1C. The PPV for s.o. PSP starts off with 73% in the 1^st^ year and continuously declines to 63.6% in the 3^rd^ year, 48.5% in the 9^th^ year, and 32.7% at the end of record. Possible and probable PSP combined according to the MDS-PSP criteria had a PPV of 87.5% in the 1^st^ year, increasing to 91% in the 3^rd^ year, before decreasing again to 81% in the 9^th^ year and ending at 81.8% at the end of record. NINDS-SPSP criteria, which are defined by a possible and probable PSP category rise in PPV from 78.3% in the 1^st^ year to 86.4% in the 3^rd^ year and then fluctuate from 85.4% in the 6^th^ year to 86.5% in the 9^th^ year back to 86.4% at the end of record. The overall PPV of the MDS-PSP criteria was 76.2% in the 1^st^ year, 71.6% in the 3^rd^, 64.7% in the 9^th^ year, and 60.8% at the end of record.

### Effect of the Exclusion Criteria on the Positive Predictive Value

We also analyzed the effect of the exclusion criteria on the PPV (Fig. 1C vs Supplementary Fig. S1C). With the omission of the exclusion criteria, PPV was reduced for all patient groups of our cohort in every year and for both the MDS-PSP and the NINDS-SPSP criteria.

### Evolution of s.o. PSP Into Higher Clinical Diagnostic Certainty Levels

**FIG. 2.**
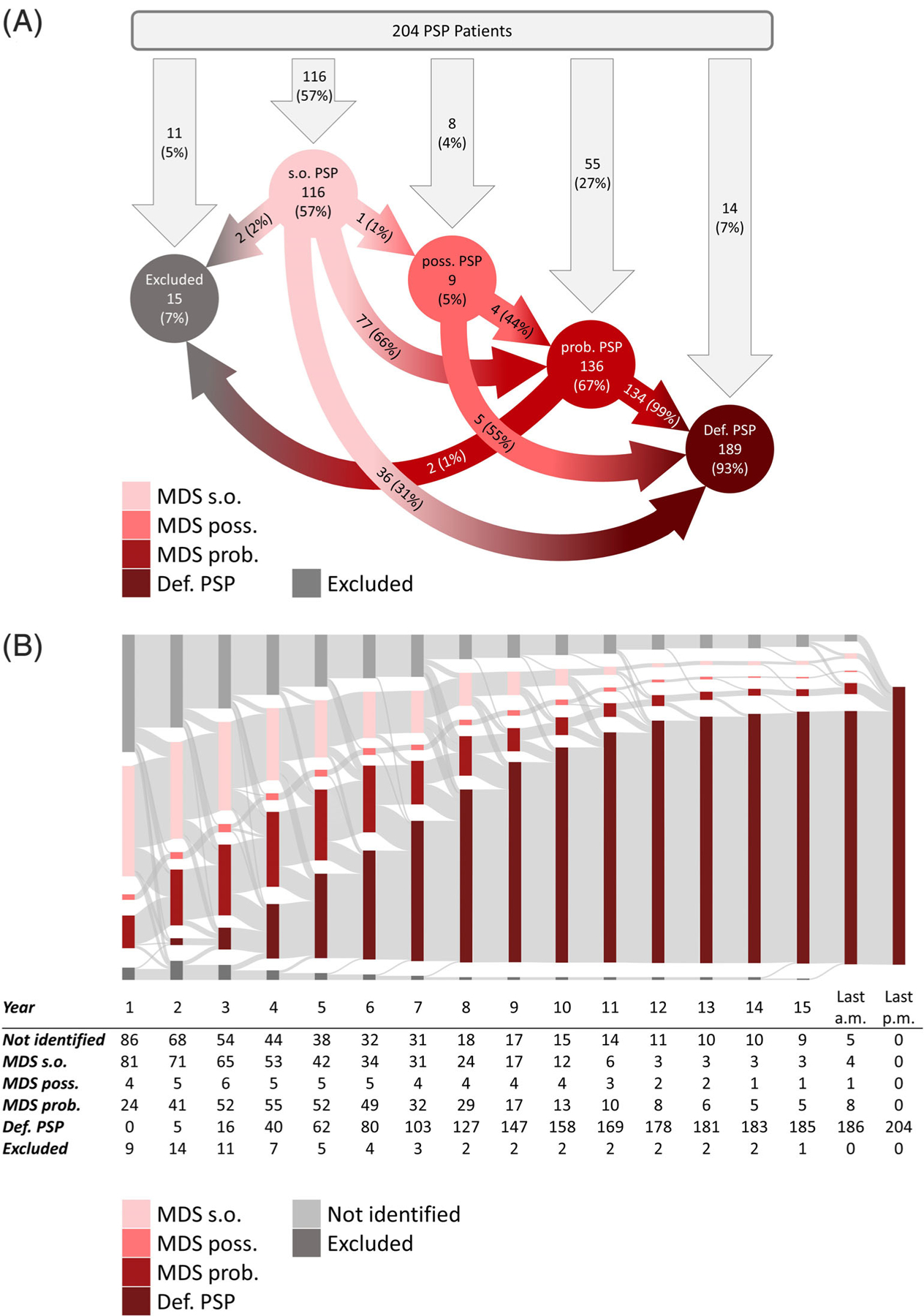
Allocation of definite PSP patients into the MDS-PSP diagnostic certainty levels. (**A**) Initial allocation and categorical evolution of diagnostic certainty levels of the MDS-PSP criteria showing the quantitative relevance of the s.o. PSP category as most frequent first clinical diagnosis in N = 204 definite PSP cases. Percentages in the arrows refer to the amount of patients in the circle at the beginning of the arrow. Definite PSP is a neuropathological diagnosis and therefore can only be stated post mortem. (**B**) Time-dependent evolution rates of diagnostic certainty levels per year of the MDS-PSP criteria showing that MDS s.o. PSP category is a highly frequent transition stage. Last a.m., last ante mortem diagnosis; last p.m., last post mortem diagnosis; Prob., probable PSP; Poss., possible PSP; S.o., suggestive of PSP; Def., definite PSP. Not identified = patients not fulfilling the diagnostic criteria; excluded = patients meeting the exclusion criteria of the clinical diagnostic criteria.

Figure 2A shows the initial allocation of the 204 autopsy confirmed PSP patients into their MDS-PSP diagnostic certainty category (downward arrows) and the evolution into higher levels of certainty (colored arrows). Only few definite PSP patients (7%, N = 14) were not identified at all by the MDS-PSP criteria before death. Few definite PSP patients (7%, N = 15) met an exclusion criterion, of which two patients were first recognized as s.o. PSP and two as probable PSP. The vast majority of cases received an initial diagnosis of s.o. PSP (57%, N = 116), followed by probable PSP (27%, N = 55), and possible PSP (4%, N = 8).

During their clinical evolution, s.o. PSP patients later met the MDS-PSP exclusion criterion of autonomic failure within the first 2 years of disease (2%, N = 2), remained s.o. PSP until final antemortem record (31%, N = 36), or evolved to possible PSP (1%, N = 1) or probable PSP (66%, N = 77).

Time-dependent evolution rates of diagnostic certainty levels per year after symptom onset in the 204 definite PSP patients are visualized in a Sankey diagram (Fig. 2B). A diagnosis of s.o. PSP was present in 40% of patients (N = 81) in the first year.

With time, the number of s.o. PSP patients continuously declined to 2% (N = 4) at the last antemortem record, mainly due to transition into definite PSP (ie, death) or probable PSP.

The majority of s.o. PSP patients (66%, N = 77) evolved into probable PSP, on average after 3.6 years. Consistently, the number of patients with probable PSP steadily increased from the first year (12%, N = 24) to a maximum in the 4^th^ year (27%, N = 55), before continuously declining again, due to the increasing number of deceased patients qualifying for definite PSP.

### Frequencies of PSP Predominance Types

**FIG. 3.**
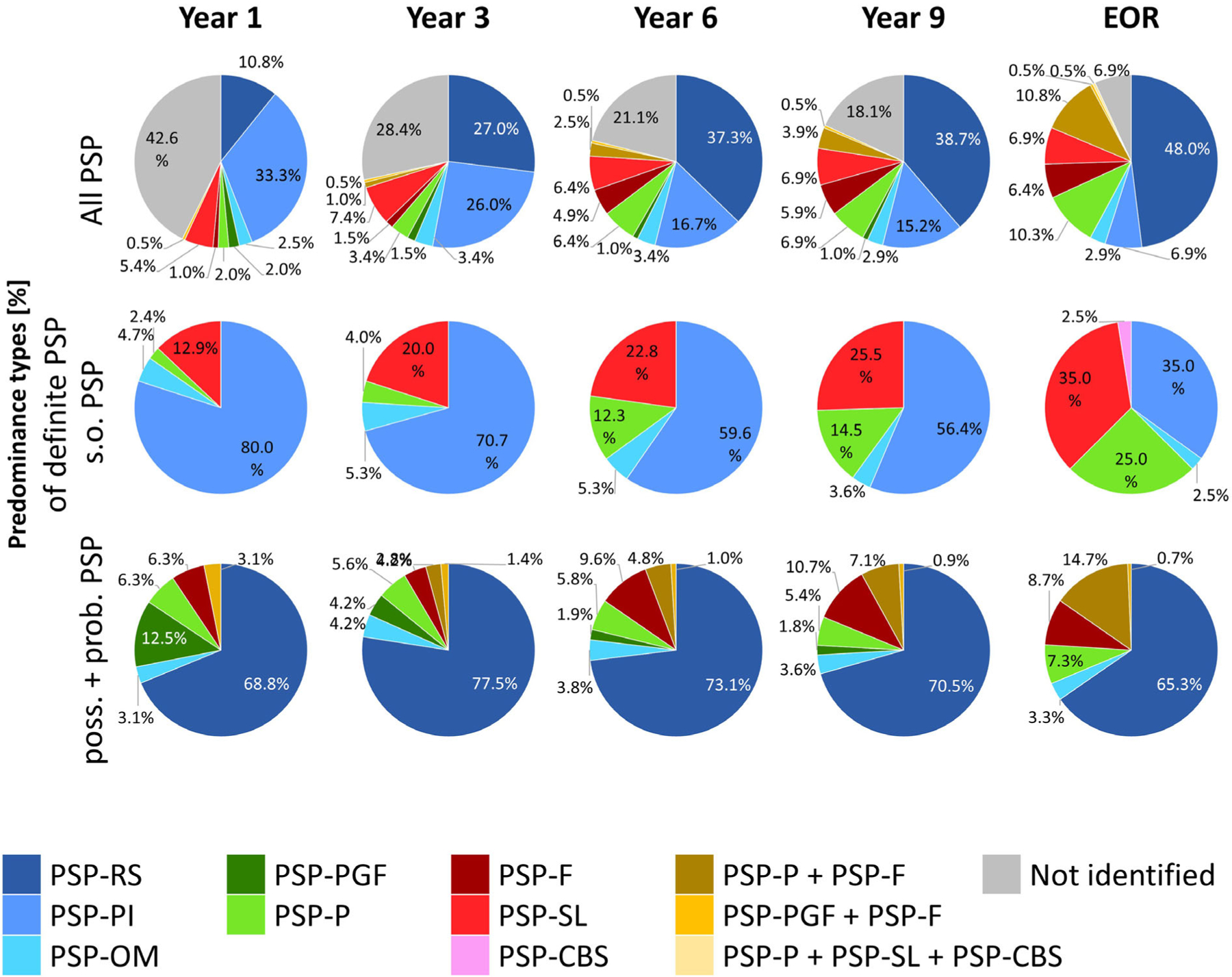
Predominance types of definite PSP patients by the MDS-criteria for the diagnosis of PSP. Data are shown as a function of disease duration (1^st^–9^th^ year) since onset of first symptoms. The top row shows all patients, the middle row patients fulfilling clinical suggestive of (s.o.) PSP criteria, the lower row patients fulfilling possible or probable PSP criteria. EOR, end of record; Prob., probable PSP; poss., possible PSP; s.o., suggestive of PSP; PSP-RS, PSP with Richardson’s syndrome; PSP-PI, PSP with predominant postural instability; PSP-OM, PSP with predominant ocular motor dysfunction; PSP-PGF, PSP with progressive gait freezing; PSP-P, PSP with predominant Parkinsonism; PSP-F, PSP with predominant frontal presentation; PSP-SL, PSP with predominant speech/language disorder; PSP-CBS, PSP with predominant corticobasal syndrome. Not identified = patients not fulfilling the diagnostic criteria.

Figure 3 shows the relative frequencies and temporal evolution of clinical predominance types in definite PSP patients for the entire cohort (top row), for the subgroups fulfilling the clinical criteria for s.o. PSP (second row), and for possible or probable PSP patients (lower row).

In the total cohort, PSP-PI was prevailing initially (1^st^ year, 33.3%), PSP-RS becoming predominant only after year 3 (3^rd^ year, 27.0%). In the s.o. PSP group, PSP-PI was the most common predominance type throughout the clinical course (1^st^ year, 80.0%; 3^rd^ year, 70.7%, and 9^th^ year, 56.4%). In possible or probable PSP patients, PSP-RS was the most frequent predominance type throughout (1^st^ year, 68.8%; 3^rd^ year, 77.5%; 9^th^ year, 70.5%).

### Transition of PSP Predominance Types

**FIG. 4.**
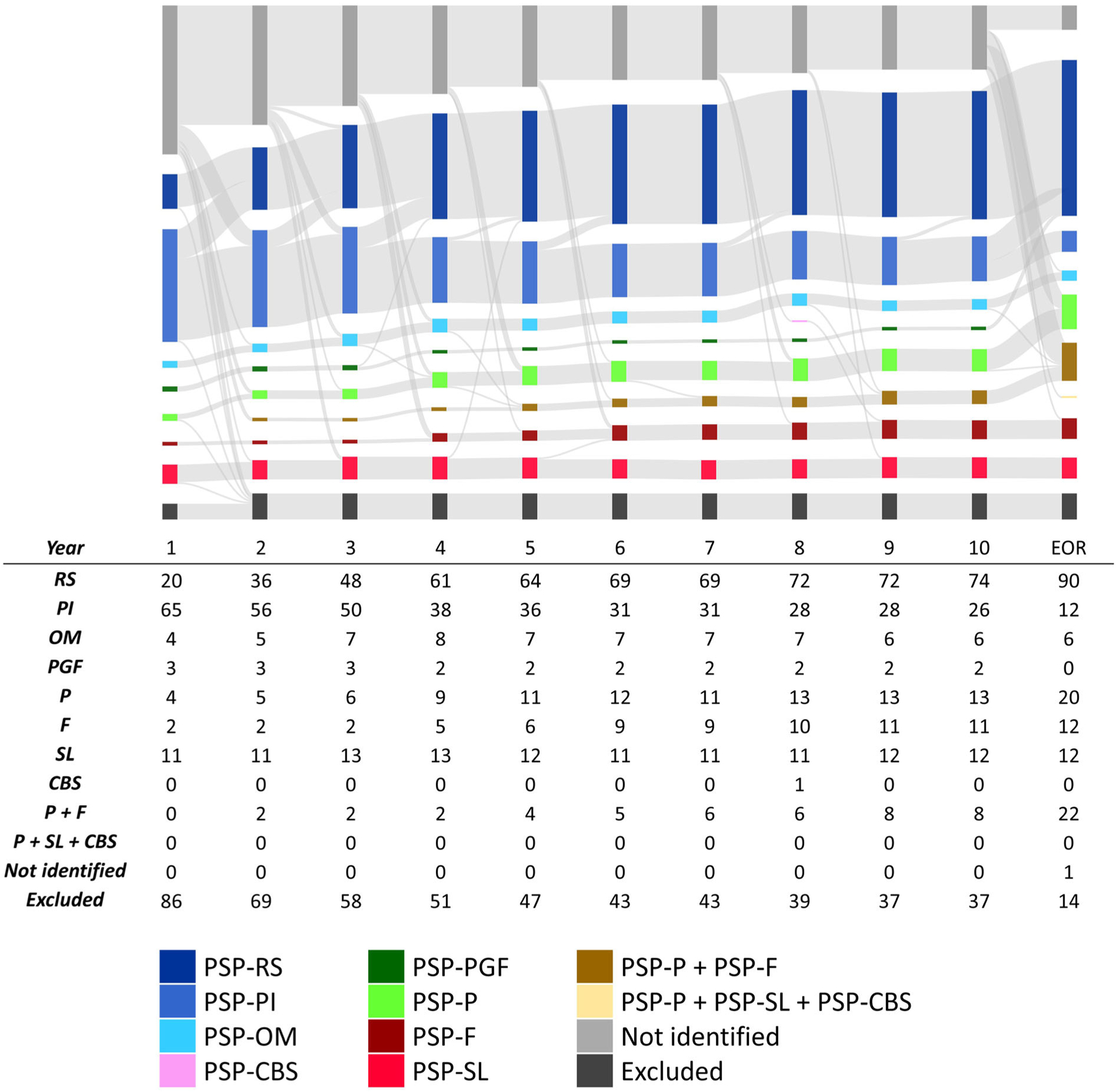
Transition of MDS predominance types between 1^st^ and 10^th^ year and at the end of record in N = 204 definite PSP patients. Each column represents 1 year, the size of the colored columns and the size of the connection strings is representative to the amount of patients. EOR, end of record; PSP-RS, PSP with Richardson’s syndrome; PSP-PI, PSP with predominant postural instability; PSP-OM, PSP with predominant ocular motor dysfunction; PSP-PGF, PSP with progressive gait freezing; PSP-P, PSP with predominant Parkinsonism; PSP-F, PSP with predominant frontal presentation; PSP-SL, PSP with predominant speech/language disorder; PSP-CBS, PSP with predominant corticobasal syndrome. Not identified = patients not fulfilling the diagnostic criteria; excluded = patients meeting the exclusion criteria of the clinical diagnostic criteria.

A detailed analysis of how patients transition between the different MDS-PSP predominance types during the disease course is shown in Figure 4. Certainty levels are disregarded in this figure.

N = 77 (38%) of all patients changed their predominance type during their clinical course, as demonstrated in detail in Supplementary Table S4. More than half (41/77; 53%) of the transitions occurred within the first 4 years. The transition from PSP-PI to PSP-RS was by far the most common (65/77; 84%). The transition from any predominance type to PSP-RS took 2.9 years on average.

### Time to Diagnosis

For the MDS-PSP criteria, the average time to first PSP diagnosis after onset of first symptoms was 2.2 ± 0.23 (range = 1–26) years using the criteria in general, 2.1 ± 0.31 (range = 1–26) years for s.o. PSP, 2.0 ± 0.44 (range = 1–4) years for possible PSP, and 3.9 ± 0.28 (range = 1–19) years for probable PSP. The combined time to diagnosis of possible and probable PSP was 3.8 ± 0.27 (range = 1–19).

For the NINDS-SPSP criteria, the average time to diagnosis was 4.0 ± 0.3 (range = 1–18) years in general, 4.7 ± 0.5 (range = 1–18) years for possible PSP, and 3.4 ± 0.4 (range = 1–15) years for probable PSP.

## Discussion

Our study shows that the novel concept of s.o. PSP contributes to the increased sensitivity of the MDS-PSP criteria for antemortem PSP diagnosis, compared to the NINDS-SPSP criteria in our cohort. The majority of PSP patients in our cohort passed through the state of s.o. PSP diagnosis (57%) and evolved to a higher level of diagnostic certainty antemortem (66%). Among those that evolved to probable or possible PSP in our cohort, the average time PSP patients spent with a diagnosis of s.o. PSP before evolving was 3.6 years. However, as the prevalence of PSP in our cohort is a lot higher than in the average neurological clinic population, further investigation with a more representative population should be assessed in future projects. S.o. PSP showed high specificity in the first 3 years of disease, declining with increasing disease duration. Application of the MDS-PSP criteria for s.o. PSP reduced the time to diagnosis by 1.6 years from 3.8 to 2.2 years on average. When compared to the NINDS-SPSP criteria, the overall time to diagnosis was reduced from 4.0 years (NINDS-SPSP) to 2.2 years (MDS-PSP).

In total, 57% (N = 116) of all PSP patients in our cohort received a diagnosis of s.o. PSP at any time until the end of record, 69.8% (N = 81) of these cases within the 1^st^ year after symptom onset and 86.2% (N = 100) within the first 3 years after symptom onset. Hence, s.o. PSP is essential to identify prospect PSP patients at an early disease stage and should play an important role for future research projects, such as clinical trials with disease modifying therapies to stop disease progression at an early stage. Considering that patients who were identified as possible or probable PSP also fulfilled s.o. PSP criteria, only 12% (N = 25) of the patients did not meet MDS-PSP criteria for s.o. PSP, either because they met exclusion criteria (5%) or they lacked necessary features (7%). These numbers need to be validated with a prospective study to see if further adjustments are possible to raise the sensitivity even more.

Sensitivity of s.o. PSP and the overall MDS-PSP criteria was lower in our study than in a previous study by Ali et al.^7^ For s.o. PSP, Ali et al^7^ found a sensitivity of 46.7% within the first 3 years of disease, compared to 34.3% in our cohort, and a sensitivity of 100% for the late stage group of >3 years disease duration, compared to 85.7% in our cohort. Sensitivity of the overall MDS-PSP criteria within the first 3 years was also lower in our cohort, with 84.4% in Ali et al,^7^ compared to 64.2% in our cohort. The fraction of patients with s.o. PSP at the end of record was very similar with 17.8% in the study by Ali et al^7^ and 17.6% in our cohort. Specificity of the overall MDS-PSP criteria was higher in our study. Within the first 3 years, specificity was 75.9%, compared to 34.2% in Ali et al.^7^ One reason for the discrepancy in specificity could be the composition of the disease control group. In the cohort of Ali et al,^7^ the control group also consisted of patients with CBD pathology, which made up 55.6% of the control group (88.9% met MDS-PSP criteria), whereas our control group consisted only 55 patients with CBD, which made up 25.5% of the entire control cohort. Moreover, most cases of the autopsy cohort presented in our study were used to develop the MDS-PSP criteria.^6^ This circularity in the analysis may have led to a general overestimation of the performance of the MDS-PSP criteria in our cohort.

The most frequent predominance type in s.o. PSP in our cohort was PSP-PI. At the same time, s.o. PSP-PI was the most frequent false–positive diagnosis in early stages in disease controls, especially in MSA-P cases. S.o. PSP-PI is defined as (1) repeated unprovoked falls within 3 years (P1), or (2) tendency to fall on the pull-test within 3 years (P2) according to the MDS-PSP criteria.^5^ In our retrospective data, details on the pull-test were not documented. Any documentation of repeated unprovoked falls within 3 years was considered as P1 and any documented postural instability within 3 years without documented falls was considered as P2. Thus, it is possible that details on performance in the pull-test would increase the number of cases diagnosed as s.o. PSP-PI. The presence of two or more steps backward on the pull test (P3) could not be assessed with our data, which is a limitation of the study. For a diagnosis of s.o. PSP-RS and s.o. PSP-F, an abnormal pull test can be decisive. Therefore, these predominance types are likely to be underrepresented in our cohort. Moreover, frequent macro square wave jerks, one of the features that characterize O_3_ were not documented. For meeting O_3_, an eyelid opening apraxia can be sufficient, however, its presence or absence was only reported in 19% of our cases and present in 9% of our cases. These likely implements an observation and recruitment bias into the distribution of the predominance types in our cohort. Adequate consideration of these features might increase sensitivity of s.o. PSP in a prospective setting, which highlights the importance of the pull-test and oculomotor evaluation in the early clinical diagnosis of PSP. Further, the high relative frequency of PSP-SL in patients with s.o. PSP underlines the importance of considering speech and language symptoms as initial presentation of PSP.

In our cohort of definite PSP patients, a majority (66%) of PSP patients diagnosed as s.o. PSP evolved into probable PSP, within an average time of 3.6 years. The evolution was mostly due to the development of abnormal saccades or supranuclear gaze palsy in patients with a diagnosis of s.o. PSP-PI, which subsequently qualified for probable PSP-RS. For interpreting the transition between different predominance types, it is crucial to keep in mind that not every predominance type is represented in every certainty level. PSP-PI is only represented in the s.o. PSP category, and therefore, disease progression makes a transition of PSP-PI to another predominance type very likely. Patients with PSP-F and PSP-P can be allocated to s.o. PSP and probable PSP. Hence, a progress in the same predominance type is possible. The high number of evolving patients emphasizes the relevance of s.o. PSP as a transition stage into higher levels of diagnostic certainty.

The number of PSP patients diagnosed as possible PSP according to the MDS-PSP criteria was very low (5%). This may be due to clinical data that could not be determined in our cohort and is one of the limitations of this study. Indeed, a diagnosis of possible PSP with PSP-RS requires the presence of P3,^5^ and thus, was not documented in our cohort. Moreover, possible PSP with predominant ocular motor dysfunction (PSP-OM) and possible PSP with progressive gait freezing (PSP-PGF) are each defined by one clinical feature only: vertical supranuclear gaze palsy (O1) and progressive gait freezing within 3 years (A1), respectively. When they are combined with other diagnostic clinical features, a diagnosis of probable PSP applies, as was the case for most of our analyzed patients. When regarding the results of our study, the design of the possible PSP category can be questioned. Further investigation in form of prospective studies will show whether the limited value of the category possible PSP is caused by the retrospective design of our study.

Specificity of the overall MDS-PSP criteria depended on the diagnostic certainty and the year after disease onset. In our cohort, s.o. PSP showed high levels of specificity within the first 3 years of disease, which declined with longer disease duration.

The PPV for possible and probable PSP according to the MDS-PSP criteria was high and superior to the PPV of the NINDS-SPSP criteria until the 3^rd^ year and remained above 80% until the end of record. The PPV for s.o. PSP decreased with increasing disease duration to 67% at end of record. This is mainly due to the increasing numbers of non-PSP cases being diagnosed with PSP-PI and PSP-SL in the early years and PSP-P in later years of the disease.

Because specificity and PPV depend on the number of cases per disease group included into the analysis, our results on specificity and PPV should be interpreted with caution. Increasing the numbers of CBD, MSA-P, or PD patients or including patients with other 4R-tauopathies, like globular glial tauopathy (GGT) or argyrophilic grain disease (AGD), would likely result in lower specificity and lower PPV. However, for therapeutic strategies that focus on tau or 4R-tau, distinguishing PSP from other 4R-tauopathies probably will not be decisive.

If the intention was to recognize any 4R-tauopathy, the specificity and PPV for both diagnostic criteria are underestimated, because the CBD patients included in our cohort significantly reduced the PPVs.

The MDS-PSP exclusion criteria excluded fewer PSP patients, but also fewer non-PSP patients, as compared to the NINDS-SPSP criteria. There are at least two reasons for this: on the one hand, “severe, asymmetric parkinsonian signs,” which are part of the NINDS-SPSP exclusion criteria, does not apply for the MDS-PSP criteria; on the other hand, our retrospective setting did not allow us to judge the severity of a symptom. Thus, we interpreted “predominant” as “present within the first 2 years of disease” for all respective MDS-PSP exclusion criteria that include this term. These are (1) “predominant, otherwise unexplained impairment of episodic memory, suggestive of Alzheimer dementia”; (2) “predominant, otherwise unexplained autonomic failure, eg, orthostatic hypotension, suggestive of multiple system atrophy, or Lewy body disease”; and (3) “predominant, otherwise unexplained visual hallucinations or fluctuations in alertness, suggestive of dementia with Lewy bodies.” Thus, in a prospective setting, “predominant” could also apply for features with later onset and lead to the exclusion of further patients. A large number of the excluded PSP patients (33.8%) developed amnestic symptoms suggestive of Alzheimer’s disease in the late disease course in our cohort. These cases met the NINDS-SPSP exclusion criteria (“cortical dementia of Alzheimer type”), but not the MDS-PSP exclusion criteria, (“predominant, otherwise unexplained impairment of episodic memory, suggestive of Alzheimer dementia”). Another 36.7% of the patients excluded by the NINDS-SPSP criteria had “severe asymmetric parkinsonian signs,” which is not an exclusion criterion of the MDS-PSP criteria. Thus, the MDS-PSP exclusion criteria do exclude less true PSP patients from a clinical diagnosis compared to the NINDS-SPSP criteria, while still maintaining an acceptable specificity. Omission of the MDS-PSP exclusion criteria would not yield a meaningful increase in sensitivity, but reduce specificity, and is therefore not recommended.

In summary, this study describes the characteristics and clinical evolution of patients diagnosed with s.o. PSP. It highlights the considerable value of s.o. PSP in identifying patients in their early clinical course, who evolve to develop possible or probable PSP and are likely to have underlying PSP pathology. Recognition of clinical conditions s.o. PSP reduce the time to first PSP diagnosis by 1.6 years on average. PSP patients remained with a diagnosis of s.o. PSP for 3.6 years on average before evolving to a higher level of diagnostic certainty. With the ultimate goal to deliver disease-modifying therapies within the earliest clinical course, it will be of high importance to further characterize patients with s.o. PSP in a prospective, real-life clinical setting and to identify biomarkers to increase the specificity of PSP or 4R-tauopathy diagnosis early on. The limitations of this study arise mainly through its retrospective nature, use of the development cohort for validation, and focus on those with known neuropathology, rather than prospective cases in community or healthcare settings. Thus, prospective validation of the diagnosis of s.o. PSP will be mandatory to verify our results.

## Supplementary Material

Supplemental Materials

## Figures and Tables

**TABLE 1.** Demographic data

	Cases	Controls				
	PSP	All	CBD	MSA	PD	FTLD
N	204	216	55	50	51	60
♂:♀ (N;[%])	107:97 [52.5:47.5]	112:104 [51.9:48.1]	107:97 [52.5:47.5]	16:34 [32.0:68,0]	25:26 [49.0:51.0]	41:19 [68.3:31.7]
Age at onset (y, mean ± SEM [range])	66.3 ± 0.6 [41–91]	59.6 ± 0.6 [42–81]	63.8 ± 1.2 [42–81]	59.7 ± 1.3 [45–80]	58.7 ± 1.5 [42–76]	56.8 ± 1.0 [42–74]
Age at death (y, mean ± SEM [range])	74.1 ± 0.6 [54–94]	68.4 ± 0.6 [47–92]	70.4 ± 1.1 [51–85]	67.1 ± 1.2 [51–90]	73.4 ± 1.2 [59–92]	63.5 ± 1.2 [47–84]
Disease duration (y, mean ± SEM [range])	7.7 ± 0.3 [0–27]	8.6 ± 0.4 [1–35]	6.8 ± 0.4 [1–12]	7.0 ± 0.3 [2–15]	14.7 ± 1.0 [3–35]	6.9 ± 0.6 [1–20]

Demographic data of autopsy-confirmed PSP patients and disease controls.

Abbreviations: CBD, corticobasal degeneration; non-4RT FTLD, non-4R-tauopathy frontotemporal lobar degeneration; MSA, multiple system atrophy; PD, Parkinson’s disease; PSP, progressive supranuclear palsy; SEM, standard error of the mean.

## Appendix

### The MDS Endorsed PSP Study Group

Ikuko Aiba, Angelo Antonini, Thomas Arzberger, Paolo Barone, Kailash P. Bhatia, Adam K. Boxer, Carlo Colosimo, Yaroslau Compta, Jean Christophe Corvol, Dennis W. Dickson, Ellen Gelpi, Armin Giese, Lawrence I. Golbe, Murray Grossman, Günter U. Höglinger, Franziska Hopfner, David J. Irwin, Keith A. Josephs, Jan Kassubek, Gabor G. Kovacs, Anthony E. Lang, Johannes Levin, Irene Litvan, Matthias Höllerhage, Nikolaus McFarland, Wassilios G. Meissner, Huw R. Morris, Ulrich Müller, Christer Nilsson, Wolfgang H. Oertel, Alexander Pantelyat, Alex Rajput, Gesine Respondek, James B. Rowe, Ruji Sakakibara, Gerard Schellenberg, Maria Stamelou, Claire Troakes, Thilo van Eimeren, John C. van Swieten, Gregor K. Wenning, Jennifer L. Whitwell.
