# Supplementary material for: Clinical Conditions “Suggestive of Progressive Supranuclear Palsy”—Diagnostic Performance
Source: Mov Disord. Author manuscript; Available in PMC 2021 Dec 1. (PMC7953080; doi:10.1002/mds.28263)
Supplement: Supplemental Materials [file NIHMS1677370-supplement-Supplemental_Materials.docx]

**Supplementary Material**

**for the article**

**Clinical conditions “suggestive of progressive supranuclear palsy” – diagnostic performance.**

Max-Joseph Grimm,^1,2*^ Gesine Respondek, MD,^2,3*^ Maria Stamelou, MD,^4,5^ Thomas Arzberger, MD,^2,6,7,^ Leslie Ferguson, MD,^8^ Ellen Gelpi, MD,^9,10^ Armin Giese, MD,^6^ Murray Grossman, MD,^11^ David J Irwin, MD,^11^ Alexander Pantelyat, MD,^12^ Alex Rajput, MD,^8^ Sigrun Roeber, MD,^6^ John C van Swieten, MD,^13^ Claire Troakes, PhD, MSc,^14^ Wassilios G Meissner, MD, PhD,^15-17^ Christer Nilsson, MD,^18^ Ines Piot,^1,2^ Yaroslau Compta, MD,^19-21^ James B Rowe, MD,PhD,^22^ Günter U Höglinger, MD,^1,2,3^ for the Movement Disorder Society-endorsed PSP Study Group. ^*^ equal contribution

**Correspondence to:** Prof. Dr. Günter U. Höglinger, Dept. of Neurology, Hannover Medical School, Carl-Neuberg-Str. 1, D-30625 Hannover, Germany. Phone: +49 511 532 2392, Fax: 49 511 532 3115, guenter.hoeglinger@dzne.de.

**Supp. Table 1:** Clinical features and their definitions.

| ***Feature*** | ***Definition*** |
| --- | --- |
| Abnormal saccades | Abnormal saccades in saccadic or smooth pursuit eye movement |
| Alien limb phenomenon | Involuntary motor activity of a limb combined with the feeling of estrangement from that limb |
| Alzheimer´s disease-like deficits | Mnestic or visuospatial deficits |
| Apraxia of limb(s) | Inability to correctly use objects or perform symbolic gestures on command |
| Apraxia of speech | A motor speech disorder characterized by slow speech rate, prolonged intervals between syllables and words, equalization of stress across syllables and words, decreased articulatory accuracy with sound distortions |
| Asymmetry at onset | Clear difference between the signs on the left and the right at the beginning of the disease |
| Asymmetry, persisting | Clear difference between the signs on the left and the right throughout the disease |
| Autonomic dysfunction | Chronic constipation, postural hypotension, sweating abnormalities, erectile dysfunction, or any two of urinary urgency, frequency or nocturia without hesitancy |
| Bradykinesia | Any mention of bradykinesia or motor slowing |
| Cerebellar signs | Intention tremor, cerebellar limb or gait ataxia, cerebellar nystagmus |
| Cognitive dysfunction | Presence of any perceived cognitive decline, including difficulty in concentration, mental slowing, or forgetfulness, reported by either the patient, relatives or the treating doctor; formal neuropsychological testing was not required |
| Cortical sensory loss | With preserved primary sensation |
| Dysarthria | Any alteration in the quality of articulation, without evidence for apraxia of speech or aphasia |
| Dysphagia | Any swallowing abnormality, severe enough to lead to aspiration of solid food or liquids |
| Dystonia, axial | Dystonia affecting neck and trunk muscles |
| Dystonia of limb(s) | Dystonia affection one or more limbs |
| Falls | Any falls without explanation other than PSP symptoms |
| Freezing of gait | Sudden stop or hesitation when passing a verge |
| Freezing of speech | Sudden stop or hesitation when speaking |
| Frontal dysfunction | Changes in personality (apathy, disinhibition, aggressiveness) and/or social skills (lack of concerns, insights, basic emotions) and/or executive skills [impairment of mental flexibility, verbal fluency, planning, problem solving, organization, attention, abstraction, judgment] and/or behavior (utilization or imitation behavior, perseveration, stereotyped behaviors, apathetic mutism, applause sign, grasp sign, palmomental reflex, snout reflex, frontal type urinary incontinence); not necessarily dementing |
| Hallucinations, spontaneous | Hallucinations not related to dopaminergic therapy |
| Hallucinations, L-dopa-induced | Hallucinations related to dopaminergic therapy |
| Levodopa-responsiveness | Improvement of >30% coincident with the introduction of levodopa |
| Myoclonus | Focal, stimulus-sensitive reflex myoclonus in a limb |
| Non-specific visual symptoms | Symptoms include painful eyes, dry eyes, visual blurring, diplopia, blepharospasm, ptosis, reduced blinking rate and eyelid apraxia |
| Postural instability | Impaired postural reflexes |
| Non-fluent/agrammatic primary progressive aphasia | Dysfunction of expressive language with severe problems in word retrieval (agrammatic, telegraphic or truncated spoken language) in the context of preserved word comprehension |
| Pyramidal tract signs | Pathologically brisk tendon reflexes or extensor plantar response |
| Rapid hypophonia | Weak or whispered voice with intact articulation early in the disease course |
| Rapid micrographia | Handwriting in increased speed and reduced size compared to previous years early in the disease course |
| Rest tremor | Tremor in a resting position |
| Rigidity, axial predominant | Rigidity affecting neck and trunk muscles more than limbs |
| Rigidity, limb predominant | Rigidity affecting limb muscles more than neck and trunk muscles |
| Semantic dementia | Impaired ability to name and understand words and to recognize the significance of faces, objects and other sensory stimuli |
| Start hesitation | Difficulty in initiating gait |
| Supranuclear gaze palsy | Restricted range of voluntary eye movement in the vertical plane with preserved vestibulo-ocular reflex |
| Tremor | Any tremor |

**Suppl. Table 2:** Transition of MDS-PSP certainty levels types between 1^st^ and 15^th^ year after symptom onset and at end of record in N=204 definite PSP patients.

|  | ***Year after symtom onset*** | | | | | | | | | | | | | | | | |  |
| --- | --- | --- | --- | --- | --- | --- | --- | --- | --- | --- | --- | --- | --- | --- | --- | --- | --- | --- |
|  | ***1*** | ***2*** | ***3*** | ***4*** | ***5*** | ***6*** | ***7*** | ***8*** | ***9*** | ***10*** | ***11*** | ***12*** | ***13*** | ***14*** | ***15*** | ***Last a.m.*** | ***Last p.m.*** | ***Total*** |
| ***n.i. -> n.i.*** | 86 | 68 | 54 | 44 | 38 | 32 | 31 | 18 | 17 | 15 | 14 | 11 | 10 | 10 | 9 | 5 | 0 |  |
| ***n.i. -> s.o.*** |  | 11 | 8 | 4 | 3 | 0 | 0 | 4 | 0 | 1 | 1 | 0 | 0 | 0 | 1 | 2 | 0 | 35 |
| ***n.i. -> poss*** |  | 1 | 1 | 1 | 0 | 1 | 0 | 0 | 0 | 0 | 0 | 0 | 0 | 0 | 0 | 0 | 0 | 4 |
| ***n.i. -> prob*** |  | 3 | 4 | 4 | 3 | 5 | 0 | 6 | 1 | 1 | 0 | 1 | 1 | 0 | 0 | 2 | 0 | 31 |
| ***n.i. -> dec*** |  | 1 | 1 | 1 | 0 | 0 | 1 | 3 | 0 | 0 | 0 | 2 | 0 | 0 | 0 | 0 | 5 | 9 |
| ***n.i. -> ex*** |  | 2 | 0 | 0 | 0 | 0 | 0 | 0 | 0 | 0 | 0 | 0 | 0 | 0 | 0 | 0 | 0 | 2 |
| ***s.o. -> s.o.*** | 81 | 60 | 57 | 49 | 39 | 34 | 31 | 20 | 17 | 11 | 5 | 3 | 3 | 3 | 2 | 2 | 0 |  |
| ***s.o. -> poss*** |  | 0 | 0 | 1 | 0 | 0 | 0 | 0 | 0 | 0 | 0 | 0 | 0 | 0 | 0 | 0 | 0 | 1 |
| ***s.o. -> prob*** |  | 17 | 11 | 13 | 9 | 6 | 3 | 6 | 3 | 3 | 2 | 2 | 0 | 0 | 1 | 1 | 0 | 77 |
| ***s.o. -> dec*** |  | 2 | 3 | 2 | 5 | 2 | 0 | 5 | 4 | 3 | 5 | 1 | 0 | 0 | 0 | 0 | 5 | 32 |
| ***s.o. -> ex*** |  | 2 | 0 | 0 | 0 | 0 | 0 | 0 | 0 | 0 | 0 | 0 | 0 | 0 | 0 | 0 | 0 | 2 |
| ***poss -> poss*** | 4 | 4 | 5 | 3 | 5 | 4 | 4 | 4 | 4 | 4 | 3 | 2 | 2 | 1 | 1 | 1 | 0 |  |
| ***poss -> prob*** |  | 0 | 0 | 2 | 0 | 0 | 0 | 0 | 0 | 0 | 1 | 0 | 0 | 1 | 0 | 0 | 0 | 4 |
| ***poss -> dec*** |  | 0 | 0 | 1 | 0 | 1 | 1 | 0 | 0 | 0 | 0 | 1 | 0 | 0 | 0 | 0 | 1 | 4 |
| ***poss -> ex*** |  | 0 | 0 | 0 | 0 | 0 | 0 | 0 | 0 | 0 | 0 | 0 | 0 | 0 | 0 | 0 | 0 | 0 |
| ***prob -> prob*** | 24 | 21 | 37 | 36 | 40 | 38 | 29 | 17 | 13 | 9 | 7 | 5 | 5 | 4 | 4 | 5 | 0 |  |
| ***prob -> dec*** |  | 1 | 4 | 16 | 15 | 14 | 20 | 15 | 16 | 8 | 6 | 5 | 3 | 2 | 1 | 0 | 8 | 126 |
| ***prob -> ex*** |  | 2 | 0 | 0 | 0 | 0 | 0 | 0 | 0 | 0 | 0 | 0 | 0 | 0 | 0 | 0 | 0 | 2 |
| ***ex -> ex*** | 9 | 8 | 11 | 7 | 5 | 4 | 3 | 2 | 2 | 2 | 2 | 2 | 2 | 2 | 1 | 0 | 0 |  |
| ***ex -> dec*** |  | 1 | 3 | 4 | 2 | 1 | 1 | 1 | 0 | 0 | 0 | 0 | 0 | 0 | 1 | 1 | 0 | 15 |
| ***dec -> dec*** |  | 0 | 5 | 16 | 40 | 62 | 80 | 103 | 127 | 147 | 158 | 169 | 178 | 181 | 183 | 185 | 204 |  |

**Suppl. Table 3:** Transition of MDS-PSP predominance types between 1^st^ an 10^th^ year after symptom onset and at end of record in N=204 definite PSP patients.

|  | | ***Year after symptom onset*** | | | | | | | | | | | | | | | | | | | | | | | |
| --- | --- | --- | --- | --- | --- | --- | --- | --- | --- | --- | --- | --- | --- | --- | --- | --- | --- | --- | --- | --- | --- | --- | --- | --- | --- |
|  | | ***1*** | | ***2*** | | ***3*** | | ***4*** | | ***5*** | | ***6*** | | ***7*** | | ***8*** | | ***9*** | | ***10*** | | ***EOR*** | | ***Total*** | |
| ***n.i. ->*** | ***n.i.*** | | 86 | | 69 | | 58 | | 51 | | 47 | | 43 | | 43 | | 39 | | 37 | | 37 | | 14 | |  |
|  | ***RS*** | | 20 | | 0 | | 2 | | 0 | | 0 | | 0 | | 0 | | 0 | | 0 | | 0 | | 0 | | 22 |
|  | ***PI*** | | 65 | | 9 | | 4 | | 0 | | 0 | | 0 | | 0 | | 0 | | 0 | | 0 | | 0 | | 78 |
|  | ***OM*** | | 4 | | 1 | | 2 | | 2 | | 0 | | 0 | | 0 | | 0 | | 0 | | 0 | | 1 | | 10 |
|  | ***PGF*** | | 3 | | 0 | | 0 | | 0 | | 0 | | 0 | | 0 | | 0 | | 0 | | 0 | | 0 | | 3 |
|  | ***P*** | | 4 | | 2 | | 1 | | 2 | | 3 | | 1 | | 0 | | 2 | | 0 | | 0 | | 8 | | 23 |
|  | ***F*** | | 2 | | 0 | | 0 | | 3 | | 1 | | 2 | | 0 | | 1 | | 0 | | 0 | | 1 | | 10 |
|  | ***SL*** | | 11 | | 1 | | 2 | | 0 | | 0 | | 0 | | 0 | | 0 | | 1 | | 0 | | 0 | | 15 |
|  | ***CBS*** | | 0 | | 0 | | 0 | | 0 | | 0 | | 0 | | 0 | | 1 | | 0 | | 0 | | 0 | | 1 |
|  | ***P + F*** | | 0 | | 2 | | 0 | | 0 | | 0 | | 1 | | 0 | | 0 | | 1 | | 0 | | 12 | | 16 |
|  | ***P + SL + CBS*** | | 0 | | 0 | | 0 | | 0 | | 0 | | 0 | | 0 | | 0 | | 0 | | 0 | | 1 | | 1 |
|  | ***Excluded*** | | 9 | | 2 | | 0 | | 0 | | 0 | | 0 | | 0 | | 0 | | 0 | | 0 | | 0 | | 11 |
| ***RS ->*** | ***RS*** | |  | | 19 | | 36 | | 48 | | 61 | | 64 | | 69 | | 69 | | 72 | | 72 | | 74 | |  |
|  | ***Excluded*** | |  | | 1 | | 0 | | 0 | | 0 | | 0 | | 0 | | 0 | | 0 | | 0 | | 0 | | 1 |
| ***PI ->*** | ***PI*** | |  | | 47 | | 46 | | 38 | | 36 | | 31 | | 31 | | 28 | | 28 | | 26 | | 12 | |  |
|  | ***RS*** | |  | | 17 | | 10 | | 12 | | 2 | | 5 | | 0 | | 3 | | 0 | | 2 | | 14 | | 65 |
|  | ***Excluded*** | |  | | 1 | | 0 | | 0 | | 0 | | 0 | | 0 | | 0 | | 0 | | 0 | | 0 | | 1 |
| ***OM ->*** | ***OM*** | |  | | 4 | | 5 | | 6 | | 7 | | 7 | | 7 | | 7 | | 6 | | 6 | | 5 | |  |
|  | ***P*** | |  | | 0 | | 0 | | 1 | | 0 | | 0 | | 0 | | 0 | | 0 | | 0 | | 0 | | 1 |
|  | ***P + F*** | |  | | 0 | | 0 | | 0 | | 1 | | 0 | | 0 | | 0 | | 1 | | 0 | | 1 | | 3 |
| ***PGF ->*** | ***PGF*** | |  | | 3 | | 3 | | 2 | | 2 | | 2 | | 2 | | 2 | | 2 | | 2 | | 0 | |  |
|  | ***RS*** | |  | | 0 | | 0 | | 1 | | 0 | | 0 | | 0 | | 0 | | 0 | | 0 | | 2 | | 3 |
| ***P ->*** | ***P*** | |  | | 3 | | 5 | | 6 | | 8 | | 11 | | 11 | | 11 | | 13 | | 13 | | 12 | |  |
|  | ***P + F*** | |  | | 0 | | 0 | | 0 | | 1 | | 0 | | 1 | | 0 | | 0 | | 0 | | 1 | | 3 |
|  | ***Excluded*** | |  | | 1 | | 0 | | 0 | | 0 | | 0 | | 0 | | 0 | | 0 | | 0 | | 0 | | 1 |
| ***F ->*** | ***F*** | |  | | 2 | | 2 | | 2 | | 5 | | 6 | | 9 | | 9 | | 10 | | 11 | | 11 | |  |
| ***SL ->*** | ***SL*** | |  | | 10 | | 11 | | 13 | | 12 | | 11 | | 11 | | 11 | | 11 | | 12 | | 12 | |  |
|  | ***F*** | |  | | 0 | | 0 | | 0 | | 0 | | 1 | | 0 | | 0 | | 0 | | 0 | | 0 | | 1 |
|  | ***RS*** | |  | | 0 | | 0 | | 0 | | 1 | | 0 | | 0 | | 0 | | 0 | | 0 | | 0 | | 1 |
|  | ***Excluded*** | |  | | 1 | | 0 | | 0 | | 0 | | 0 | | 0 | | 0 | | 0 | | 0 | | 0 | | 1 |
| ***CBS ->*** | ***CBS*** | |  | | 0 | | 0 | | 0 | | 0 | | 0 | | 0 | | 0 | | 0 | | 0 | | 0 | |  |
|  | ***F*** | |  | | 0 | | 0 | | 0 | | 0 | | 0 | | 0 | | 0 | | 1 | | 0 | | 0 | | 1 |
| ***P + F ->*** | ***P + F*** | |  | | 0 | | 2 | | 2 | | 2 | | 4 | | 5 | | 6 | | 6 | | 8 | | 8 | |  |
| ***Excluded ->*** | ***Excluded*** | |  | | 9 | | 15 | | 15 | | 15 | | 15 | | 15 | | 15 | | 15 | | 15 | | 15 | |  |

**Suppl. Table 4:** Number of cases of PSP, CBD, MSA, LBD and non-4RT FTLD and coresponding brain banks.

|  | ***PSP*** | ***CBD*** | ***MSA*** | ***LBD*** | ***Non-4RT FTLD*** | ***Total*** |
| --- | --- | --- | --- | --- | --- | --- |
| ***Balt*** |  | 12 | 7 | 10 |  | 29 |
| ***Barc*** | 27 | 10 | 10 | 11 |  | 58 |
| ***Bord*** |  |  | 7 | 5 |  | 12 |
| ***Lond*** | 32 | 14 | 2 | 5 |  | 53 |
| ***Lund*** | 9 |  |  |  |  | 9 |
| ***Munc*** | 61 | 15 | 19 | 15 | 22 | 132 |
| ***Penn*** | 4 | 4 |  |  | 32 | 40 |
| ***Rott*** | 38 |  | 5 | 5 | 6 | 54 |
| ***Sask*** | 33 |  |  |  |  | 33 |
| ***Total*** | 204 | 55 | 50 | 51 | 60 | 420 |

Abbreviations: *Balt*: Johns Hopkins Medical Institutions (JHMI) Brain Resource Center, Baltimore, USA; *Barc*: Neurological Tissue Bank of the Biobanc-Hospital Clinic-IDIBAPS, Barcelona, Spain in collaboration with the Neurology Department of the Hospital Clinic; *Bord*: Centre de Ressources Biologiques – Bordeaux Biothèque Santé, Bordeaux, France; *Lond*: MRC London Neurodegenerative Diseases Brain Bank, King´s College, London, UK; *Lund*: Brain Bank of Region Skane, Department of Pathology, Lund University, Lund, Sweden; *Munc*: Neurobiobank Munich, Center for Neuropathology and Prion Research, Ludwig-Maximilians-University, Munich, Germany.; *Penn*: Penn Center for Neurodegenerative Disease Research (CNDR) brain bank, University of Pennsylvania, Philadelphia, USA; *Rott*: Netherlands Brain Bank, Amsterdam in collaboration with the Department of Neurology, Erasmus Medical Center, Rotterdam, The Netherlands; *Sask*: Brain Bank of the Royal University Hospital, University of Saskatchewan, Canada; *PSP:* progressive supranuclear palsy; *CBD:* corticobasal degeneration; *MSA:* multiple system atrophy; *LBD:* Lewy body dementia; *non-4RT FTLD:* non-4R-tauopathy frontotemporal lobar degeneration;

**Supplementary Figure 1: Sensitivity, specificity, and positive predictive value with omission of the exclusion criteria.**


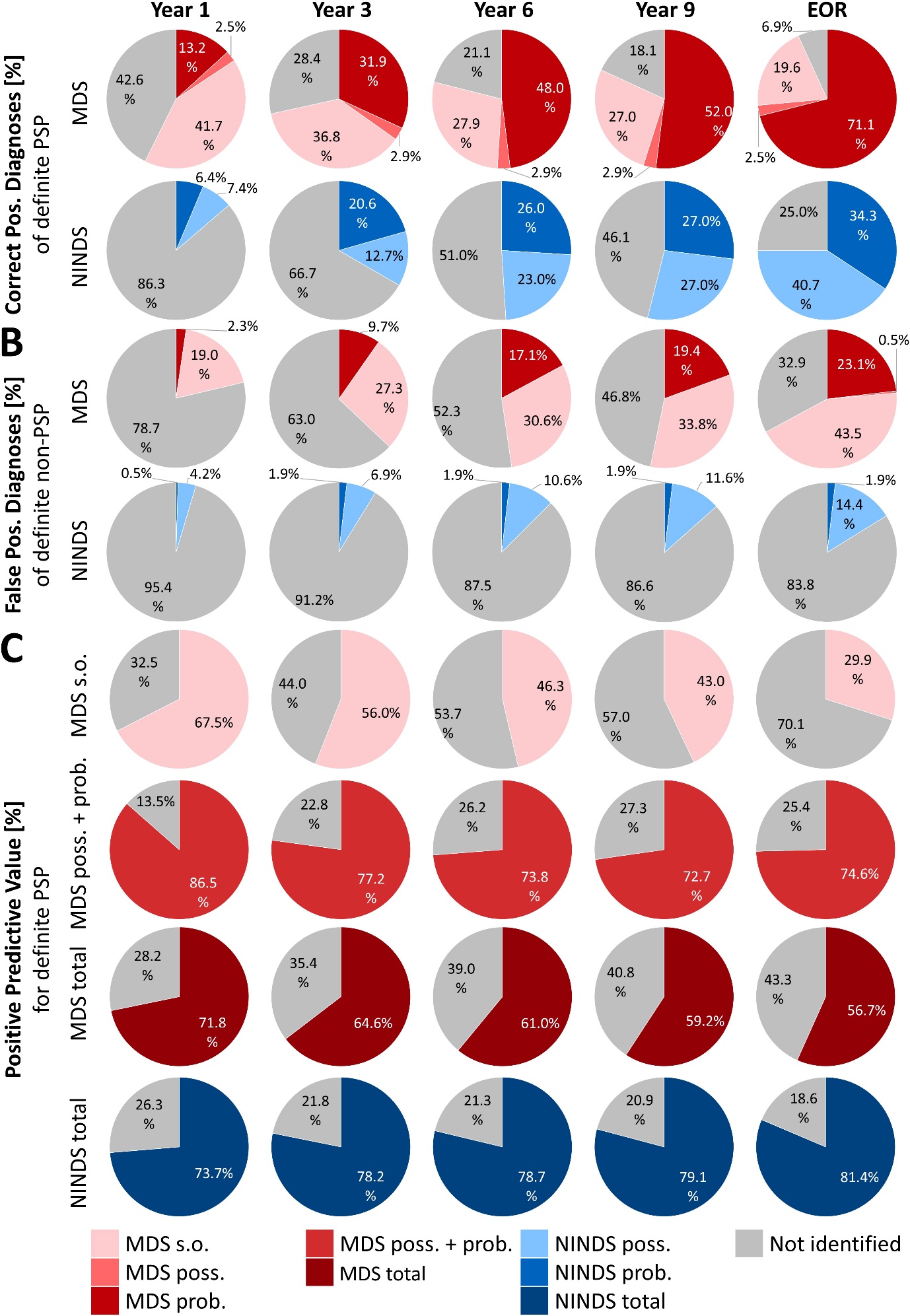


(**A)** Rate of correct positive PSP diagnoses in definite PSP (i.e. sensitivity) and (**B**) false positive PSP diagnoses in non-4R-tauopathies (i.e. 100% - specificity) with the MDS-PSP criteria (probable, possible or suggestive of PSP) and NINDS-SPSP criteria (probable or possible PSP) as a function of disease duration (1^st^ – 9^th^ year) since onset of first symptoms.

**(C)** Positive predictive value (PPV) for s.o. PSP (first row), possible or probable PSP combined (second row), and PSP of all certainty levels combined (third row) according to the MDS-PSP criteria; and of all certainty levels combined according to the NINDS-SPSP criteria (possible and probable PSP; forth row).

EOR = end of record. prob. = probable PSP. poss. = possible PSP. s.o. = suggestive of PSP. Not identified = patients not fulfilling the respective clinical diagnostic criteria.

**Supplementary Figure 2: False positive diagnoses in distinct disease entities, with application of the exclusion criteria.**


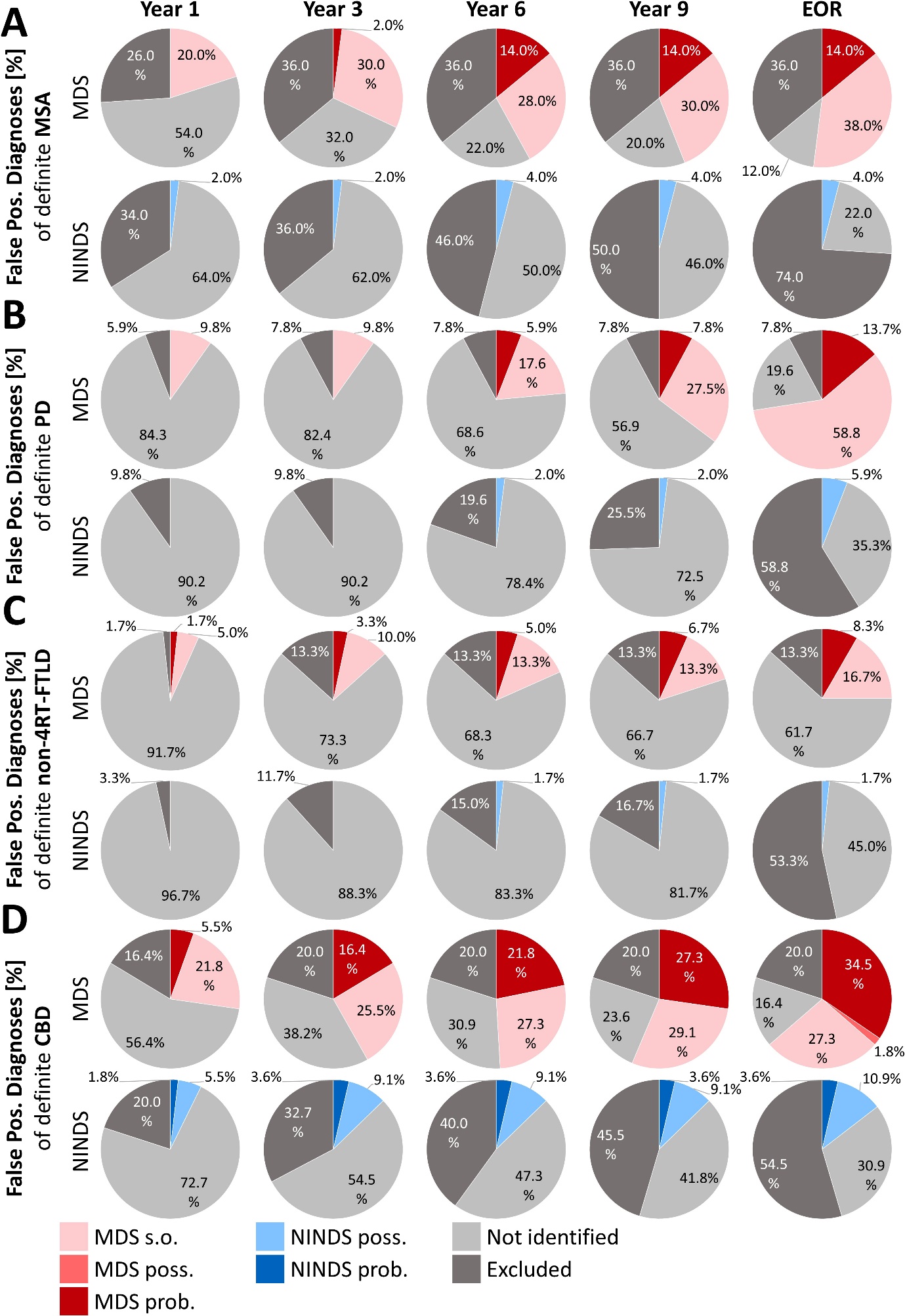


Rate of false positive PSP diagnoses in MSA (**A**), PD (**B**), non-4RT-FTLD (**C**) and CBD (**D**) with the MDS-PSP and NINDS-SPSP criteria, respectively, as a function of disease duration (1^st^ – 9^th^ year) since onset of first symptoms.

EOR = end of record. Not identified = patients not fulfilling the respective clinical diagnostic criteria. Excluded = patients meeting the exclusion criteria of the respective clinical diagnostic criteria.

**Supplementary Figure 3: False positive diagnoses in distinct disease entities, with omission of the exclusion criteria.**


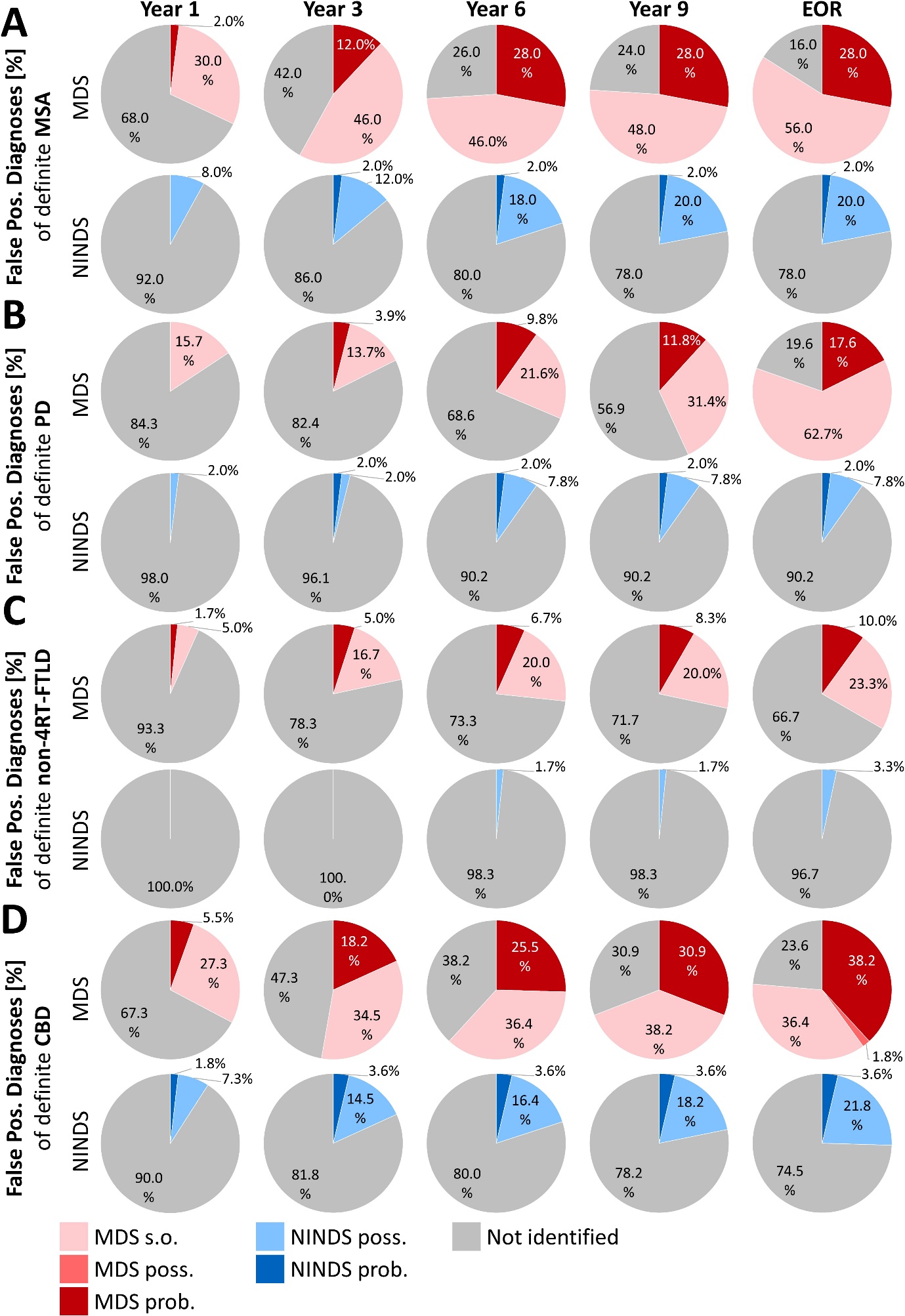


Rate of false positive PSP diagnoses in MSA (**A**), PD (**B**), non-4RT-FTLD (**C**) and CBD (**D**) with the MDS-PSP and NINDS-SPSP criteria, respectively, as a function of disease duration (1^st^ – 9^th^ year) since onset of first symptoms.

EOR = end of record. Not identified = patients not fulfilling the respective clinical diagnostic criteria. Excluded = patients meeting the exclusion criteria of the respective clinical diagnostic criteria.
